# Newborn screening for X-linked adrenoleukodystrophy in Italy: Diagnostic algorithm and disease monitoring

**DOI:** 10.3389/fneur.2022.1072256

**Published:** 2023-01-09

**Authors:** Eleonora Bonaventura, Luisella Alberti, Simona Lucchi, Laura Cappelletti, Salvatore Fazzone, Elisa Cattaneo, Matteo Bellini, Giana Izzo, Cecilia Parazzini, Alessandra Bosetti, Elisabetta Di Profio, Giulia Fiore, Matilde Ferrario, Chiara Mameli, Arianna Sangiorgio, Silvia Masnada, Gian Vincenzo Zuccotti, Pierangelo Veggiotti, Luigina Spaccini, Maria Iascone, Elvira Verduci, Cristina Cereda, Davide Tonduti, XALD-NBS Study Group, Gianluca Lista

**Affiliations:** ^1^Child Neurology Unit, V. Buzzi Children's Hospital, Milan, Italy; ^2^Center for Diagnosis and Treatment of Leukodystrophies and Genetic Leukoencephalopathies (COALA), V. Buzzi Children's Hospital, Milan, Italy; ^3^Newborn Screening and Inherited Metabolic Disease Unit, V. Buzzi Children Hospital, Milan, Italy; ^4^Clinical Genetics Unit, V. Buzzi Children's Hospital, Milan, Italy; ^5^Molecular Genetics Laboratory, ASST Papa Giovanni XXIII, Bergamo, Italy; ^6^Paediatric Radiology and Neuroradiology Department, V. Buzzi Children's Hospital, Milan, Italy; ^7^Department of Paediatrics, V. Buzzi Children's Hospital, University of Milan, Milan, Italy; ^8^Department of Biomedical and Clinical Sciences, University of Milan, Milan, Italy; ^9^Department of Health Sciences, University of Milan, Milan, Italy

**Keywords:** X-ALD, X-linked adrenoleukodystrophy (X-ALD), Zellweger Spectrum Disorders, Aicardi-Goutières syndrome (AGS), hematopoietic stem cell transplantation (HCST), newborn screening (NBS), DBS, C26:0-lysophosphatidylcholine

## Abstract

**Introduction:**

X-linked adrenoleukodystrophy (X-ALD) is the most common inherited peroxisomal disorder caused by variants in the *ABCD1* gene. The main phenotypes observed in men with X-ALD are primary adrenal insufficiency, adrenomyeloneuropathy, and cerebral ALD (cALD). Cerebral ALD consists of a demyelinating progressive cerebral white matter (WM) disease associated with rapid clinical decline and is fatal if left untreated. Hematopoietic stem cell transplantation is the standard treatment for cALD as it stabilizes WM degeneration when performed early in the disease. For this reason, early diagnosis is crucial, and several countries have already implemented their newborn screening programs (NBS) with the assessment of C26:0-lysophosphatidylcholine (C26:0-LPC) values as screening for X-ALD.

**Methods:**

In June 2021, an Italian group in Lombardy launched a pilot study for the implementation of X-ALD in the Italian NBS program. A three-tiered approach was adopted, and it involved quantifying the values of C26:0-LPC and other metabolites in dried blood spots with FIA-MS/MS first, followed by the more specific ultra-performance liquid chromatography-tandem mass spectrometry (UHPLC-MS/MS) technique and, finally, the genetic confirmation *via* focused NGS.

**Discussion:**

Genetically confirmed patients are set to undergo a follow-up protocol and are periodically evaluated to promptly start a specific treatment if and when the first signs of brain damage appear, as suggested by international guidelines. A specific disease monitoring protocol has been created based on literature data and personal direct experience.

**Conclusion:**

The primary aim of this study was to develop a model able to improve the early diagnosis and subsequent follow-up and timely treatment of X-ALD.

**Ethics:**

The study was approved by the local ethics committee. The research was conducted in the absence of any commercial or financial relationship that could be construed as a potential conflict of interest.

## Introduction

X-linked adrenoleukodystrophy (X-ALD) is the most common inherited peroxisomal disorder, with an estimated prevalence of 1/15.000–17.000 (1). It consists of an inborn error of very long-chain fatty acids (VLCFA) beta-oxidation caused by variants in the X chromosome ATP binding cassette subfamily D member 1 (*ABCD1*) gene. This gene encodes a peroxisomal transmembrane protein necessary for the entrance of VLCFA into the peroxisome where they are digested and eliminated (2, 3). A defect in ABCD1 protein leads to increased concentrations of the VLCFA in tissues, including the brain, the spinal cord, and the adrenal cortex, and in the plasma (4, 5).

The main phenotypes observed in men with X-ALD are primary adrenal insufficiency (Addison disease), adrenomyeloneuropathy (AMN), and cerebral ALD (cALD), alone or in any combination. Addison's disease occurs in the first decade of life with a lifetime prevalence of 80–90% (6). Demyelinating progressive cerebral white matter (WM) disease with rapid clinical declining (cALD) occurs in about 35% of boys with *ABCD1* mutation, most often during the first decade of life (3–10 years) and turns fatal if untreated (7). Most men who do not develop cALD during childhood develop AMN in adult life. In this latter case, AMN is clinically characterized by spastic paraparesis, sensory-motor peripheral neuropathy, sphincter disturbances, and sexual dysfunction as the main symptoms (5).

On the other hand, female carriers of pathogenetic variants of *ABCD1* (in up to 88% of cases) develop a milder and later phenotype of AMN, with peripheral neuropathy or myelopathy as the main manifestation (8). Central involvement or adrenal insufficiency is not commonly observed in women (<1%) (3).

Early diagnosis, therefore, is crucially important. Hematopoietic stem cell transplantation (HSCT) is the standard treatment for cALD. It is not able to recover WM degeneration but it leads to its stabilization, and as a consequence, the earlier it is performed, the better the final outcome (9). Given the significant associated morbidity and mortality related to HSCT, and considering that only some men with X-ALD develop cALD, this is only recommended for patients with an active cerebral disease diagnosed at an early stage (Loes score <10 and no neurological signs) (10, 11). As WM lesions on magnetic resonance imaging (MRI) precede clinical neurological manifestations, their early recognition offers the opportunity to intervene in a pre-symptomatic stage of the disease.

Gene therapy is also being studied as an alternative if allogeneic donor options are poor, but it is not yet available for routine care (12).

Most X-ALD men also develop Addison's disease during their lifetime, which is associated with high morbidity and mortality. The early identification of mutated *ABCD1* among men with X-ALD is therefore essential to monitor adrenal hormone levels and start hormone replacement therapy when needed (13).

Given the fundamental importance of early diagnosis of X-ALD, over the past ten years, several countries have implemented their newborn screening (NBS) program with the assessment of C26:0-lysophosphatidylcholine (C26:0-LPC) values in dried blood spot (DBS) for screening X-ALD. The first state to introduce this screening was the state of New York in 2013 (14), and since February 2016, X-ALD has been added to the United States Recommended Uniform Screening Panel (RUSP). Since then, NBS for X-ALD has been implemented in other states of the USA (15–21). Preliminary studies have been conducted in China and India to promote its implementation (22, 23). In the Netherlands, a pilot study (the SCAN study) has been set up recently to develop a gender-specific screening for X-ALD (24). Indeed, the Dutch Health Council recommended ALD screening only for men because “symptoms in women usually develop later and are untreatable” (*Health Council of Netherlands 2015*) (24). Inspired by the New York state's three-tier screening algorithm, the Dutch ALD screening is based on four tiers: (1) the quantification of C26:0-LPC by FIA-MS/MS in both men and women, (2) the “X-Counter” to distinguish boys from girls genetically, (3) the quantification of C26:0-LPC by the more specific HPLC-MS/MS technique (only in male newborns), and (4) the *ABCD1*gene sequencing (24).

The Italian NBS program currently includes 49 conditions and is conducted between 48 and 72 h after birth. A review of the diseases included in the Italian NBS is carried out every 2 years according to the evolution of scientific evidence on rare genetic diseases. In June 2021, we launched a pilot study for the implementation of X-ALD in the Italian NBS program. To our knowledge, this is the first project on newborn screening for X-ALD that was officially started in Italy.

C26:0-LPC can be positive in other peroxisomal disorders (PD), including Zellweger Spectrum Disorders, *ACOX1, HSD1B4, ACBD5* deficiency, and CADDS (Contiguous*ABCD1 DXS1357/BCAP31* Deletion Syndrome). These conditions can therefore be considered the cause of “false positivity” in X-ALD screening in NBS. Peroxisomal disorders constitute a group of severe neurodegenerative conditions that can present at all ages, usually with progressive severe neurological abnormalities due to the central and peripheral nervous system involvement associated with ophthalmological abnormalities, sensorineural hearing loss, hepato-digestive problems, kidney involvement, and adrenocortical insufficiency. MRI images exceedingly show the coexistence of both developmental abnormalities and progressive WM degeneration. Treatment is essentially symptomatic, and hematopoietic stem cell transplantations have been proposed anecdotally as a possible therapeutic option (25). Recently, it was demonstrated that another leukodystrophy, namely Aicardi-Goutières Syndrome (AGS), can be detected early through C26:0-LPC (14, 26). AGS is the prototype of leukodystrophy with cerebral calcification. The most common clinical presentations are neonatal and infantile AGS. Symptoms of congenital AGS are microcephaly severe jitteriness, irritability, muscular tone and movement abnormalities, poor feeding, and possible seizures. The onset of the infantile form at around 4 months of age occurs with less specific signs: irritability, unexplained fevers, sleep-wake disorders, feeding difficulties, followed by a loss of motor skills, the appearance of spastic-dystonic tetraplegia, and intellectual disability. To date, JAK 1/2 Inhibitors and Reverse-Transcriptase Inhibitors seem to be promising therapeutic options. Preliminary results in a small subset of patients have shown encouraging results (27–29).

The primary aim of our study was to develop a model for a regional NBS program to be able to offer to all newborns X-ALD screening as a part of the general NBS program and therefore improve the early diagnosis and subsequent follow-up and timely treatment of X-ALD. In this pilot phase, we decided to evaluate the possibility that some of the false positive subjects could be affected by PD or AGS considering the important therapeutic implication, namely early diagnosis and early symptomatic care in PD and early access to new therapeutic options in AGS. Therefore, secondary targets of the project are the early identification, follow-up, and treatment of those with PD and AGS that tested “false positive” by X-ALD-NBS.

## Algorithm for X-ALD newborn screening

The pilot study presented in this report is ongoing (started in June 2021 and will end in June 2024).

So far, the Neonatal Care Units from 33 hospitals in the Italian region of Lombardy have joined the program, led by a multidisciplinary team from V. Buzzi Children's Hospital in Milano. The clinical component of this group consists of three child neurologists, two geneticists, two pediatric endocrinologists, one pediatrician expert in child nutrition, one dietician, three pediatric neuroradiologists, and two physical therapists.

The program integrates the expertise of two labs: the laboratory of Newborns Screening at V. Buzzi Children's Hospital, which is the reference lab for NBS in Lombardy since 1977, and the Medical Genetics lab of Papa Giovanni XXIII Hospital in Bergamo, which is one of the main reference centers for the genetic diagnosis of rare diseases in Italy.

### Ethics approval and informed consent

The study was centrally approved by the Ethical Committee of Milano area 1 (2020/ST/395).

X-ALD neonatal screening program was proposed and explained to the parents of all newborns with gestational age ≥ 37 + 0 born in Lombardy. Both male and female newborns were included in the study. Adherence to screening for X-ALD was voluntary and participation in this study was based on informed consent signed by both parents.

A separate written consent specific for genetic analysis was proposed in case of positive tests for the first and the second tier screening.

### Sample collection and biochemical analysis

**Figure 1:**
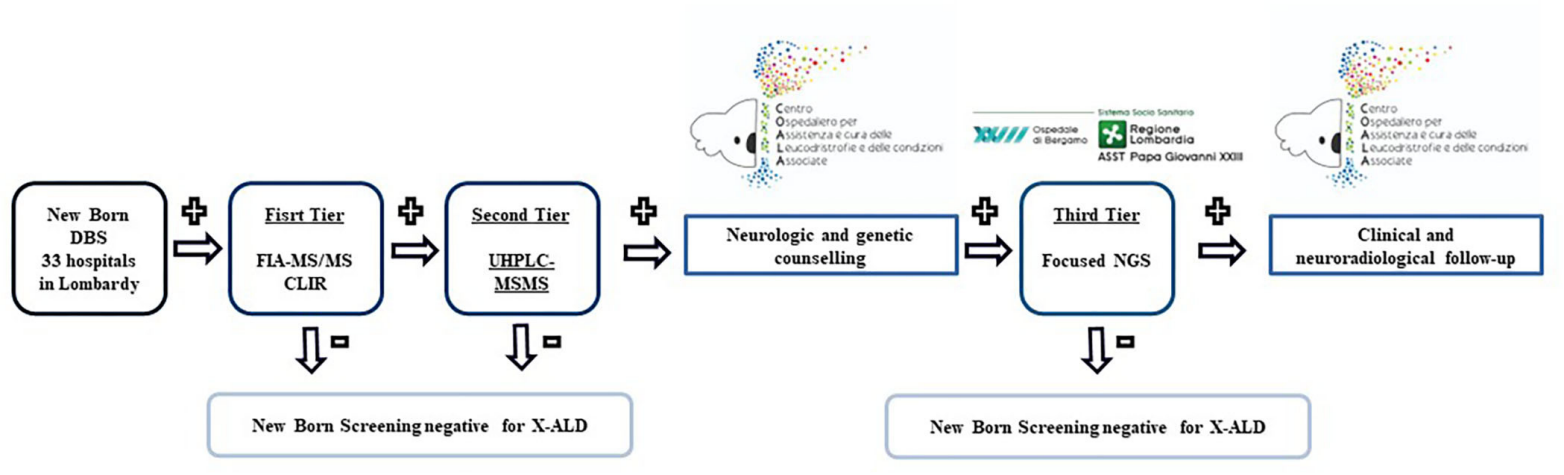
Diagnostic process of X-ALD newborn screening.

A three-tiered approach is adopted (Figure 1). DBS specimens for X-ALD screening are collected from the newborns using the standard heel prick method. DBS cards are then sent to the Newborn Screening and Metabolic Diseases Unit at V. Buzzi Children's Hospital for analysis.

The screening for X-ALD is based on the quantification of not only C20:0-LPC, C22:0-LPC, C24:0-LPC, C26:0-LPC but also C20:0-, C22:0-, C24:0-, and C26:0-acylcarnitine.

The first tier involves the measurement of these metabolites with FIA-MS/MS and the selection of a “non-negative” sample is done using a web-based post-analytical tool in Collaborative Laboratory Integrated Reports (CLIR) and the cut-offs determined from our reference population (samples of newborn ≥ 37 W.G.E. obtained between 48 and 72 h of life). In the first month of the study, reference intervals and cut-off values were analyzed in 1,000 anonymous DBS samples already available in the lab.

The analysis of C20:0-LPC, C22:0-LPC, C24:0-LPC, C26:0-LPC, and C20:0-, C22:0-, C24:0-, and C26:0-acylcarnitine are performed using the Neobase 2 newborn screening kit (PerkinElmer) following the manufacturer's instructions. Single 3.2 mm disks are punched with DBS and transferred into 96-well plates. A total of 125 mL of the PerkinElmer Neobase 2 extraction working solution (EWS) is added to each well. The microplate is covered with an adhesive microplate cover and shaken for 30 min at 650–750 rpm at 45°C. The microplate cover is removed and 100 mL was transferred to a new microplate and covered with an adhesive microplate cover before being analyzed with a PerkinElmer QSight 210. According to our reference population, the first tier is considered “non-negative” if C:26:0-LPC values, measured along with FIA-MS/MS, are greater than or equal to 0.5 uM.

Ultra-performance liquid chromatography-tandem mass spectrometry (UHPLC-MS/MS) is performed as a second-tier test on the same well as the first tier. Analysis is done using a QSight MD UHPLC Pump system (Perkin Elmer) consisting of a binary solvent manager, a vacuum degasser, a column heater, and a sample manager. About 5 μL of the extract is injected into a Gemini 3 μm C6-Phenyl 110 Å 100 mm x 2.0 mm column from Phenomenex (Torrance, CA, USA). Metabolites are separated by a linear gradient between solution A (0.1% formic acid in H2O) and solution B (0.1% formic acid in acetonitrile). The UHPLC run is 15 min at a flow rate of 0.4 mL/min. All gradient steps are linear and as follows: at T = 0 min: 70% B, toward T= 10 min: 100% B; T=10–13 min 100% B isocratic, and T = 13–15 min back to 70% B. For the mass spectrometric detection, a QSight 225 MD (Perkin Elmer) is used in the positive electrospray ionization mode with the following parameters: ESI voltage: 5200 V, HSID 320°C, Nebulizer gas: 120 L/H, Drying gas: 90L/H, source: 325°C. For the acylcarnitines, the following multiple reaction monitoring (MRMs) is used: C26:0-carnitine (540.50 > 85.00) and D4-C26:0-carnitine (544.50 > 85.00), both using a dwell time of 115 ms, CCL2-96 V and a collision energy of 44 eV. For C26:0-LPC, the following MRMs were used: C26:0-lysoPC (636.60 > 104.10) and D4- C26:0-LPC (640.60 > 104.10), both using a dwell time of 100 ms, CCL2−112 V and collision energy of 46 eV. C26:0-carnitine and C26:0-LPC levels were calculated using Simplicity software. According to our reference population, the second tier is considered positive if C26:0-LPC values, measured along with UHPLC-MS/MS, are >0.1 uM.

### Genetic counseling and gene sequencing

The newborns who test positive also at the second tier screening are referred to C.O.A.L.A. (Center for the diagnosis and treatment of leukodystrophies and genetic leukoencephalopathies) at V. Buzzi Children's Hospital in Milan, where multidisciplinary counseling with neurologists and geneticists took place first. A second written informed consent for specific genetic analyses is collected after providing families with specific information regarding X-ALD, its possible manifestations over time, and its dissimilar impact on different sexes. More specifically related to female newborns, the importance of the investigation is emphasized for the possible identification of other affected male babies and not for an effective possibility of therapeutic intervention on the newborn. If the family agrees to these investigations, focused NGS is performed first on the patient sample and then, if positive, on the parental samples for segregation analysis.

Venous blood samples are collected and sent for analysis to the Laboratory of Medical Genetics at Papa Giovanni XXIII Hospital in Bergamo. Introns and exons of the *ABCD1* gene are sequenced from genomic DNA extracted from blood in both male and female infants. If this is negative, the analysis is extended to the Zellweger Spectrum Disorders and Aicardi-Goutières syndrome genes (see Table 1).

**Table 1:** Peroxisomal and Aicardi-Goutières syndrome genes.

**X-linked adrenoleukodystrophy**
*ABCD1*
**Aicardi-Goutières syndrome**
*TREX1*
*RNASEH2B*
*RNASEH2A*
*RNASEH2C*
*SAMHD1*
*ADAR1*
*IFIH1*
*LSM11*
*RNU7-1*
*RNASET2*
**Zellweger Spectrum Disorders**
*HSD17B4*
*SCP2*
*ACOX1*
*ACBD5*
*DNM1L*
*PEX1*
*PEX2*
*PEX3*
*PEX5*
*PEX6*
*PEX10*
*PEX11B*
*PEX12*
*PEX13*
*PEX14*
*PEX16*
*PEX19*
*PEX26*

Briefly, whole-genome sequencing (WGS, Illumina DNA PCR-Free kit) is performed on a Novaseq 6,000 by 150 base pair paired-end reads. To create the sequencing files targeted only to the genomic regions of interest, the WGS bam file (generated by Dragen v 3.0 Illumina) is cut by Samtols to develop a smaller bam file encompassing only the target region. The bam file is then reconverted in fastq while all original fastQ are deleted. Ultimately, the raw file (fastq) containing only the target region is obtained. The small and specific bam file is re-run with Dragen to get the vcf file, which was annotated and filtered. A bioinformatics pipeline in bash language is created to minimize errors and speed up the process.

Variants in the *ABCD1* gene are evaluated using the Adrenoleukodystrophy Variant Database (https://adrenoleukodystrophy.info), the Human Gene Mutation Database (www.hgmd.cf.ac.uk), and published literature. Variants are also assessed for their frequency in the general population as reported in public databases (e.g., gnomAD.broadinstitute.org) and for their predicted effect. The American College of Medical Genomics guidelines are used to classify the identified variants (29).

A diagnosis of X-ALD is considered confirmed if a known pathogenic variant, likely pathogenic variant, or VUS are detected in the *ABCD1* gene.

Genetically confirmed patients will undergo a second neuro-genetic counseling to discuss the results of the analyses; an extended family screening to identify other family members with the same variant is also proposed.

## X-ALD follow-up

The results of the genetic investigations are obtained within one week of the collection of the samples.

Then, genetically confirmed patients are set to undergo a follow-up protocol and are periodically evaluated to promptly start a specific treatment if and when the first signs of brain damage appear, as suggested by international guidelines.

**Figure 2:**
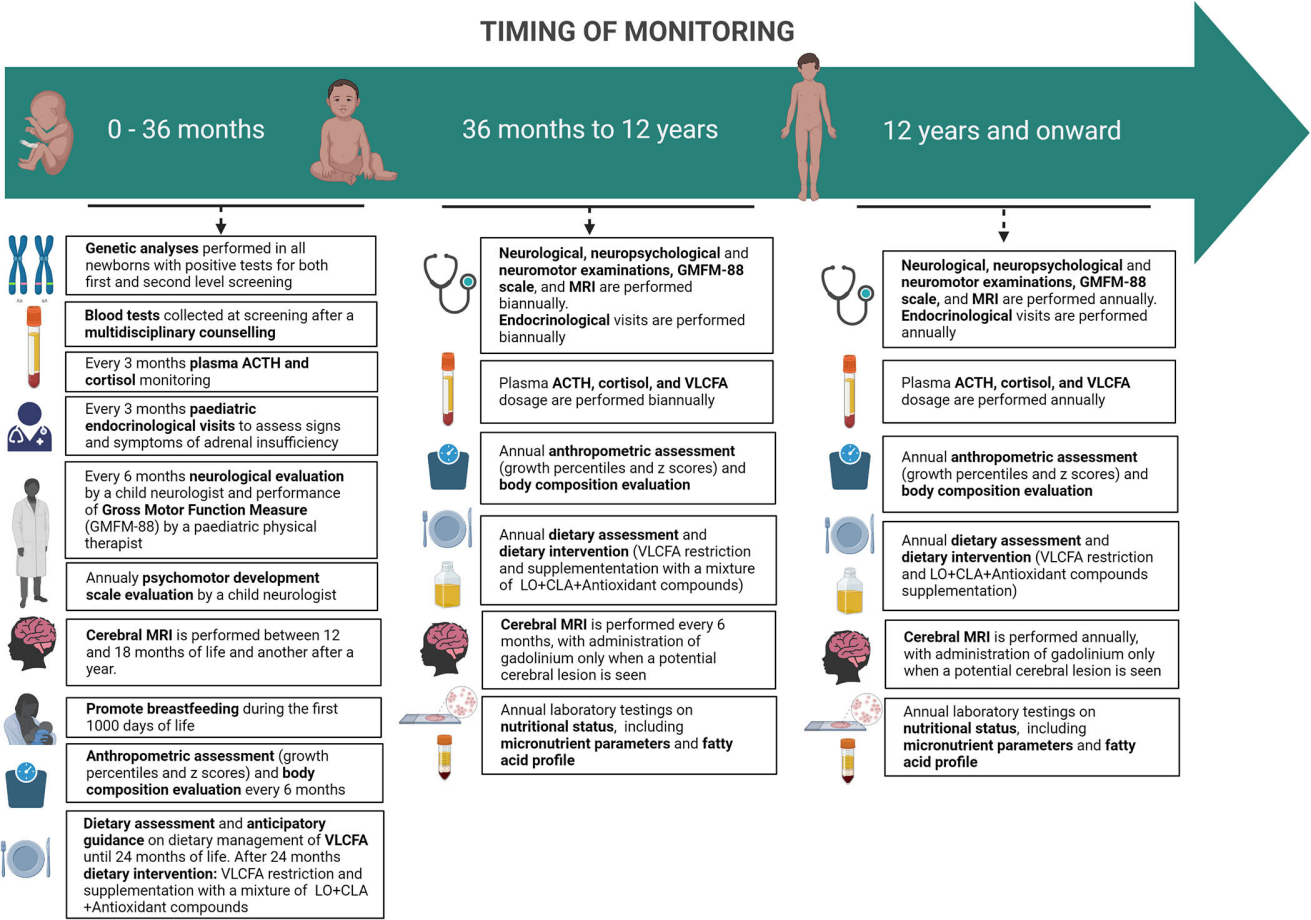
Timing of monitoring of pre-symptomatic ABCD1-mutated males subjects.

A specific disease monitoring protocol has been created based on literature data and personal direct experience (Figure 2).

### Neuromotor assessment

From 0 to 12 years, a neurological evaluation by a child neurologist and the Gross Motor Function Measure (GMFM-88) by a pediatric physical therapist is set to be performed every 6 months. In addition, in the first 42 months of life, patients undergo a psychomotor development scale evaluation (Bayley Scales of Infant and Toddler Development—Third Edition) by a child neurologist and then substituted by an age-appropriate full intellectual evaluation through the age-appropriate Wechsler Intelligence Scales.

The same assessments are set to be carried out annually from the age of 12 onwards.

### Endocrinological assessment

In the first 24 months of life, pediatric endocrinological visits are scheduled to take place every 3 months to assess signs and symptoms of adrenal insufficiency; plasma ACTH and cortisol are also monitored quarterly. When the diagnosis is confirmed, basal serum cortisol and ACTH are performed as soon as possible. Within 6 months from birth, samples of ACTH and cortisol may be taken randomly, considering that diurnal secretion has not been established yet. After 6 months from birth hormonal sample should be taken at 8 am. If a normal adrenal function is detected (ACTH <100 pg/ml and cortisol ≥5 mcg/dl), hormonal exams should be repeated in 3 months. When ACTH is between 100 and 299 pg/ml, with a cortisol <10 mcg/dl, adrenal insufficiency cannot be ruled out and a high-dose ACTH test must be performed. An ACTH ≥300 pg/ml and cortisol <18 mcg/dl are indicative of adrenal insufficiency and glucocorticoid replacement therapy must begin (30).

From 2 to 12 years of age, children should follow the same schedule every four to 6 months (30) and after 12 years of age annually (12).

### Nutritional assessment and treatment

A pediatric nutritional visit is scheduled within 3 months from birth after neonatal screening by a specialized nutritional team composed of a nutritional pediatric doctor, dietitians, and biologists that is part of the multidisciplinary group involved in the follow-up of patients with X-ALD. Subsequently starting with a complementary feeding period, patients' anthropometric parameters are to be recorded every 6 months, along with dietary assessment until 36 months of life.

From 36 months to 12 years, nutritional and anthropometric assessments are to be performed annually. Thereafter, during adolescence, the same assessments are to be carried out annually.

#### Dietary intervention

The dietary management of X-ALD has three main focuses: the restriction of Very Long Chain Fatty Acids (VLCFA) with emphasis on avoidance of C26:0 (hexacosanoic acid), the inhibition of fatty acids elongation, and the enhancement of peroxisomal beta-oxidation by the administration of a mixture of Lorenzo's oil (LO) [a 4:1 mixture of glyceryl trioleate (GTO) (C18:1 n-9) and glyceryl trierucate (GTE) (C22:1 n-9)], conjugated linoleic acid (CLA), and antioxidants compounds.

Considering the crucial role of dietary lipids for growth and neurodevelopment in the early stages of life, no fat intake restrictions are suggested for patients until 2 years of life, but foods with naturally low content VLFCA are suggested (see Table 2). During this period of life, a balanced diet according to Dietary Reference Values (DRV) is provided to the family (32). Dietary fats restriction starts after two years of life, and the diet aims to reduce C26:0 intake to <2–3 mg/day (33). Thus, fat intake through food is <15% of the total calories. Families are instructed on the main foods to be avoided or selected according to their C26:0 content (31). Particularly, attention has to be paid concerning fatty food and to the constituent of the cut in the outer covering of plants, fruits, vegetables, and nuts (31, 33) (see Table 2).

**Table 2:** Content of C26:0 (mg) in 100 g of different edible foods.

**Fish, meat, egg, dairy, and legumes**		**Pasta, bread, and cereals**		**Fruits and vegetables**		**Fats and oils**	
**Low content (<0.5 mg/100 g)**		**Low content (<0.5 mg/100 g)**		**Low content (<0.5 mg/100 g)**		**Low content (<0.5 mg/100 g)**	
Egg whites	0.007	Potato, peeled, boiled	0.096	Green apple, peeled	0.045	Medium-chain triglyceride oil	0.450
Milk, skim	0.050	Commercial breakfast cereal	0.220	Mushrooms, fresh, boiled	0.055		
Chicken, breast, skinned, broiled	0.060	Rice, long grain, cooked	0.270	Carrots, peeled	0.061		
Flounder, fresh, broiled	0.068	Flour, white	0.320	Strawberries, fresh, deseeded	0.064		
Ham, lean, fat trimmed, boiled	0.072			Cantaloupe, fresh	0.066		
Steak, fat trimmed, broiled	0.083			Tomato, peeled, deseeded	0.071		
Sausage, lean, grilled	0.084			Cherries, fresh, peeled	0.077		
Beef, ground round, broiled	0.120			Eggplant, fresh, peeled, deseeded, cooked	0.088		
Cottage cheese, 1%	0.120			Turnips, fresh, peeled, cooked	0.088		
Pork chops, extra lean, fat trimmed	0.130			Peach, fresh, with peel	0.091		
Green pees, frozen, cooked	0.180			Green pepper, fresh	0.092		
Turkey, breast, skinned, broiled	0.220			Strawberries, fresh, with seeds	0.094		
Yogurt, strawberry	0.470			Purple plum, fresh, with peel	0.160		
Cod, fresh, broiled	0.480			Watermelon	0.160		
				Butternut squash, cooked	0.190		
				Tomato, fresh, with skin and seeds	0.290		
				Green grapes, fresh, peeled	0.310		
				Lettuce hearts	0.370		
				Broccoli, frozen, boiled	0.450		
**Medium content (0.5–1 mg/100 g)**		**Medium content (0.5–1 mg/100 g)**		**Medium content (0.5–1 mg/100 g)**		**Medium content (0.5–1 mg/100 g)**	
Pinto beans, fresh, soaked and cooked	0.590	Bread, homemade	0.700	Green apple with peel	0.500		
Shrimp, fresh, broiled	0.760			Spinach, frozen, cooked	0.510		
Tofu	0.850			Apricots, dried, stewed	0.710		
				Banana	0.780		
**High content (**>**1 mg/100 g)**		**High content (**>**1 mg/100 g)**		**High content (**>**1 mg/100 g)**		**High content (**>**1 mg/100 g)**	
Beef frank	1.23	Rye bread	1.02	Green beans, string, canned	1.48	Milk, chocolate, low fat	3.33
		White bread, commercial brand, nocrust	1.39	Cabbage, fresh, uncooked	2.19	Safflower oil	5.57
		White bread, commercial brand, with crust	1.48	Zucchini, fresh, skinned, cooked	2.87	Coconut oil	5.99
		Commercial French fries, frozen, baked	2.29			Sunflower oil	8.81
		All grain commercial breakfast cereal	5.63			Olive oil	19.9
		Potato chips	7.46			Corn oil	31.3
						Peanut oil	208.4

Modified from Van Duyn et al. (31).

Simultaneously, a mixture of LO, CLA, and antioxidant compounds is to be administered orally in a dosage that provides ~20% of total caloric intake. Patients can either take trioleate glycerol (GTO) separately or incorporate it into food during meals to replace margarine, butter, mayonnaise, and cooking oils (34, 35). Considering pre-clinical studies (36–38) and clinical outcomes reported in ALD female carriers (39), the mixture containing LO, CLA, and antioxidants is a promising therapeutic approach targeted to contrast VLCFA accumulation and to reduce inflammatory markers, although more extensive studies are required.

Multivitamin and mineral supplements are prescribed according to individual needs, with particular attention to dietetic intakes of Vitamin D and B12. To avoid essential fatty acid deficiency, eicosapentaenoic acid (EPA) or docosahexaenoic acid (DHA) intakes are evaluated (35, 40).

#### Laboratory parameters

Since children with neurological impairment are at major risk of having poor micronutrient status, the following laboratory tests are to be performed annually as part of the nutritional assessment: vitamin A, B12, D (25-OH), E, folic acid, iron, ferritin, calcium, zinc, and phosphorus (41). Other blood laboratory testing includes serum albumin, prealbumin, urea, creatinine, and glucose (see Table 3). Considering the possible side effects of Lorenzo's oil on platelet levels (mild platelet anisocytosis), it is important to evaluate the mean corpuscular volume (42).

**Table 3:** Laboratory tests for nutritional assessment.

**Standard blood tests**	**Guthrie test**
Hemoglobin, mean corpuscular volume, ferritin, iron	Analysis of fatty acids (FA) profile, particularly A
Calcium, magnesium, phosphate and zinc	
Vitamins A, B12, D, E, folic acid	
VLCFA blood levels	
Glucose	
Creatinine	
Total protein	
Albumin, prealbumin	
Liver enzymes	
Urea and electrolytes	

Guthrie test paper is used to collect whole blood for the analysis of fatty acids (FA) profile, particularly docosahexaenoic acid (DHA). FA profile is evaluated in a drop of blood collected on a Guthrie paper embedded with butylated hydroxytoluene (BHT) as an antioxidant and stored in a refrigerator until analysis. Later, FA methyl esters are analyzed by gas chromatography using a GC-2100 (Shimadzu Italia S.r.l., Milano, Italy) equipped with a 15 m capillary column (DBB Agilent), PTV injector, and FID detection.

### Neuroradiological assessment

A cerebral MRI is performed every 6 months between 2 and 12 years and annually from the age of 12 onwards. MRI studies are performed on a 3 Tesla scanner (MAGNETOM VIDA, SIEMENS), with a protocol including T1, T2, and FLAIR sequences (see Table 4), in accordance with the consensus guidelines for the imaging surveillance of asymptomatic X-ALD patients (12, 43). For the studies performed before 3 years of age, the use of Gadolinium is not recommended because the likelihood of developing an active, inflammatory brain lesion remains low (44). Advanced Imaging sequences like Diffusion Tensor Tractography (DTI) or spectroscopy are not recommended in the routine clinical surveillance protocol; we decided to include them as a part of a research endeavor.

**Table 4:** MRI protocol.

	**Sequences**
Standard	3D-T1-GRE
	TSE-T2_AX
	TSE-T2_COR
	STIR-T2_SAG
	FLAIR_AX
	FLAIR_SAG
	DWI_AX
	SWI_AX
	(DTI)
	(Spectroscopy)
	STIR-T2 SAG SPINE
	TSE-T1 SAG SPINE
Lesion	TSE-T1 SAG SPINE post-Gad
	3D-T1-GRE post-Gad
	AX T1-SE post-Gad

From 3 to 12 years, imaging guidelines recommend the use of contrast-enhanced brain MRI every 6 months, as the likelihood of developing an active brain lesion is highest in this age group. To limit the exposure of patients to repeated contrast, we perform a real-time MRI reading with the administration of gadolinium only when a potential cerebral lesion is seen in agreement with the literature (12, 45). If a lesion or questionable lesion is seen, then GAD is applied. The same assessments are carried out annually from the age of 12 onwards. After this age, the use of gadolinium is limited to those cases with evidence of a cerebral lesion on a previous MRI or when there is a clinical concern.

In the case of a first lesion in an asymptomatic patient, before proceeding to HSCT, MRI is repeated after 3 months to exclude an arrested cALD (12).

MRI is evaluated through a standardized severity score (Loes score) which measures the location and extent of WM lesions and the presence of local or global atrophy (46). Each area is scored as 0 if normal, 0.5 in case of unilateral involvement, and 1 if the lesion or atrophy is bilateral. According to the literature, values ≤9 are considered early Loes scores within which HSCT should be performed (9). More specifically, in the early stages, cALD lesions are characterized by small T2 hyperintensities most often involving the genu or the splenium of the corpus callosum and may not exhibit clear gadolinium enhancement (47); alternatively, some authors reported a potential self-arrest of a subset of early lesions (48). In these cases, an MRI is repeated in 3 months to reassess the signal alteration or for gadolinium enhancement whose presence indicates active cALD and represents the need for HSCT. If a more prominent, clearly gadolinium-enhancing early cALD lesion is detected, the patient is triaged for HSCT.

### Female carriers of ABCD1

Female carriers of *ABCD1* pathogenetic variants typically develop a milder and later phenotype and symptoms are usually untreatable. For these reasons, no regular follow-up at a pediatric age is proposed. Considering the absence of a specific treatment, an early diagnosis in these cases could be a major source of parental stress. However, genetic data are still important for identifying and eventually treating other family members with the same variant and for counseling in case of further pregnancies.

To investigate the level of parental stress related to X-ALD newborn screening and to find the best way to assist and support parents in the process of diagnosis, in addition to the pilot study presented here, another study based on parent interviews is currently underway. Moreover, families are offered the possibility of consulting a psychologist who collaborates with C.O.A.L.A.

## Expected outcome

About 70,000 neonates are born in Lombardy every year. Excluding premature infants (around 10% of newborns) and newborns from families and Neonatal Units that refuse to participate in the study, we expect to include at least 40,000 newborns each year. Given the prevalence of X-ALD (1:17.000), other peroxisomal diseases (1:50.000), direct experience with AGS (no epidemiological data available), and other published pilot studies (15–21), we expect to select 10 “non-negative patients” per year. We expect that genetic analyses will confirm the results of NBS in 80% of patients, who will be immediately recruited in the surveillance protocol.

We aim to validate the NBS program linked to the clinical monitoring program and eventually investigate the optimal therapeutic strategies.

## Conclusion

Early diagnosis of X-ALD allows for monitoring and timely therapeutic intervention in children who would otherwise die from progressive and irreparable cerebral damage.

Considering the evidence of the efficacy of HSCT in arresting disease progression of cerebral phenotype, when performed in the early stage of the disease, the introduction of X-ALD into NBS would significantly change the natural history of the disease in these patients. Furthermore, early diagnosis would allow for endocrinological monitoring and early intervention, as Addison's disease is frequent and potentially lethal in X-ALD.

The results from our pilot study and those conducted internationally will constitute the basis on which the X-ALD screening can be introduced into the current Italian NBS program to offer all newborns early diagnosis, follow-up, and timely treatment to affected patients.

## Ethics statement

The studies involving human participants were reviewed and approved by the Ethical Committee of Milano area 1 (2020/ST/395). Written informed consent to participate in this study was provided by the patients/participants' legal guardian/next of kin.

## XALD-NBS study group

Gianluca Lista and Paola Fontana: Department of Neonatology and Neonatal Intensive Care Unit, V. Buzzi Children's Hospital, ASST Fatebenefratelli-Sacco, Milan, Italy. Tiziana Varisco and Olivia Casati: Department of Paediatrics, Desio Hospital, ASST Brianza, Monza, Italy. Alberto Fabio Podestà and Maddalena Gibelli: Department of Paediatrics and Neonatology, San Carlo Borromeo Hospital, ASST Santi Paolo e Carlo, Milan, Italy. Stefano Martinelli and Roberta Restelli: Neonatology and Neonatal Intensive Care Unit, Niguarda Hospital, Milan, Italy. Laura Maria Pogliani and Roberta Agistri: Department of Paediatrics, Neonatology and Neonatal Pathology, Legnano Hospital, ASST Ovest MI, Milan, Italy. Marco Giuseppe Nedbal and Paolo Vaglia: Department of Paediatrics and Neonatology, Gallarate Hospital, ASST Valleolona, Milan, Italy. Chryssoula Tzialla and Luisa Magnani: Department of Paediatrics and Neonatology, Oltrepò Pavese Hospital, ASST Pavia, Pavia, Italy. Elena Sala and Laura Lorioli: Department of Neonatal Pathology, ASST Papa Giovanni XXIII, Bergamo, Italy. Giuseppe Banderali and Diana Ghisleni, Clinical Department of Neonatology, San Paolo Hospital, ASST Santi Paolo e Carlo, Milan, Italy. Bruno Drera and Marta Frittoli: Department of Neonatal Pathology, ASST Cremona, Cremona, Italy. Francesca Lizzoli and Marta Bellini: Department of Paediatrics, Neonatology and Neonatal Pathology, Magenta Hospital, ASST Ovest Milanese, Milan, Italy. Paola Bruni and Ilaria Giulini, Department of Paediatrics, ASST Melegnano-Martesana, Milan, Italy. Valentina Benedetti and Valentina Polimeni: Department Neonatology and Neonatal Intensive Care Unit, IRCCS Ca' Granda Ospedale Maggiore Policlinico, Milan, Italy. Nadia Salvoni and Masotina Raffaele: Department Neonatology and Neonatal Pathology, Sacco Hospital, ASST Fatebenefratelli-Sacco, Milan, Italy. Cristina Bellan and Roberto Bottino: Department Neonatology and Neonatal Intensive Care Unit, Bolognini Hospital, ASST Bergamo Est, Bergamo, Italy. Graziano Barera and Antonella Poloniato: Neonatal Unit, San Raffaele Scientific Institute, Milan, Italy. Marta Odoni and Ilaria Dalla Verde: Department of Paediatrics and Neonatology, Policlinico San Pietro, Gruppo Ospedaliero San Donato, Bergamo, Italy. Massimo Agosti and Angela Bossi: Department of Paediatrics, Neonatology and Neonatal Intensive Care Unit, ASST Settelaghi, Varese, Italy. Anna Tosi and Anna Elisabetta Bussolini: Department of Paediatrics, Tradate Hospital, ASST Settelaghi, Varese, Italy. Francesco Maria Risso and Vania Spinoni: Department of Neonatology and Neonatal Intensive Care Unit, Children Hospital, ASST Spedali Civili, Brescia, Italy. Nicola Altamura and Patrizia Ballista: Department of Paediatrics and Neonatology, Sesto San Giovanni Hospital, ASST Nord Milano, Milan, Italy. Silvia Di Chio and Luciana Pagani: Department of Neonatal Pathology and Neonatal Intensive Care Unit, Macedonio Melloni Hospital, ASST Fatebenefratelli-Sacco, Milan, Milano. Lidia Decembrino and Michela Grignani: Department of Paediatrics and Neonatology, Vigevano Hospital, ASST Pavia, Pavia, Italy. Grazia Morandi and Valeria Angela Fasolato: Department of Neonatology and Neonatal Intensive Care Unit, Carlo Poma Hospital, ASST Mantova, Mantova, Italy. Lorella Rossi and Emilio Palumbo: Department of Paediatrics and Neonatology, Eugenio Morelli Hospital, ATS Montagna, Sondrio, Italy. Alessandro Lepore and Maria Forestieri: Department of Paediatrics and Neonatology, Busto Arsizio Hospital, ASST Valleolona, Varese, Italy. Stefano Ghirardello and Elisa Civardi: Department of Neonatal Pathology and Neonatal Intensive Care Unit, San Matteo Research Hospital, Pavia, Italy. Paolo Adamoli: Department of Paediatrics, Moriggia Pelascini Hospital, Gravedona, Italy. Roberta Giacchero: Department of Paediatrics and Neonatology, ASST Lodi, Lodi, Italy. Giovanni Traina: Department of Paediatrics, ASST-Melegnano Martesana, Melzo, Italy. Salvatore Barberi: Department of Paediatrics, Rho Hospital, ASST-Rhodense, RHO, Milan, Italy. Patrizia Calzi and Fenesia Pedace: Department of Neonatology, Carate Brianza Hospital, ASST Brianza, Carate, Italy. Marco Sala: Department of Paediatrics, Vimercate Hospital, Vimercate, Italy.

## Author contributions

Study conceived by: EB, LA, MF, SM, LS, MI, EV, CC, and DT. Drafting of the manuscript: EB, LA, GI, CP, ED, GF, MF, AS, LS, MI, EV, CC, and DT. All authors critical revision of the manuscript for important intellectual content, read, and approved the final manuscript.
