# Corrigendum: Newborn screening for X-linked adrenoleukodystrophy in Italy: diagnostic algorithm and disease monitoring

**DOI:** 10.3389/fneur.2024.1376447

**Published:** 2024-03-06

**Authors:** Eleonora Bonaventura, Luisella Alberti, Simona Lucchi, Laura Cappelletti, Salvatore Fazzone, Elisa Cattaneo, Matteo Bellini, Giana Izzo, Cecilia Parazzini, Alessandra Bosetti, Elisabetta Di Profio, Giulia Fiore, Matilde Ferrario, Chiara Mameli, Arianna Sangiorgio, Silvia Masnada, Gian Vincenzo Zuccotti, Pierangelo Veggiotti, Luigina Spaccini, Maria Iascone, Elvira Verduci, Cristina Cereda, Davide Tonduti, XALD-NBS Study Group, Gianluca Lista

**Affiliations:** ^1^Child Neurology Unit, V. Buzzi Children's Hospital, Milan, Italy; ^2^Center for Diagnosis and Treatment of Leukodystrophies and Genetic Leukoencephalopathies (COALA), V. Buzzi Children's Hospital, Milan, Italy; ^3^Newborn Screening and Inherited Metabolic Disease Unit, V. Buzzi Children Hospital, Milan, Italy; ^4^Clinical Genetics Unit, V. Buzzi Children's Hospital, Milan, Italy; ^5^Molecular Genetics Laboratory, ASST Papa Giovanni XXIII, Bergamo, Italy; ^6^Paediatric Radiology and Neuroradiology Department, V. Buzzi Children's Hospital, Milan, Italy; ^7^Department of Paediatrics, V. Buzzi Children's Hospital, University of Milan, Milan, Italy; ^8^Department of Biomedical and Clinical Sciences, University of Milan, Milan, Italy; ^9^Department of Health Sciences, University of Milan, Milan, Italy

**Keywords:** X-ALD, X-linked adrenoleukodystrophy (X-ALD), Zellweger Spectrum Disorders, Aicardi-Goutières syndrome (AGS), hematopoietic stem cell transplantation (HCST), newborn screening (NBS), DBS, C26:0-lysophosphatidylcholine

In the published article, there was an error in the XALD-NBS study group list, and authors Stefano Ghirardello, Elisa Civardi, Paolo Adamoli, Roberta Giacchero, Giovanni Traina, Salvatore Barberi, Patrizia Calzi, Fenesia Pedace, and Marco Sala were erroneously excluded. The corrected XALD-NBS study group section is below.

## XALD-NBS study group

Gianluca Lista and Paola Fontana: Department of Neonatology and Neonatal Intensive Care Unit, V. Buzzi Children's Hospital, ASST Fatebenefratelli-Sacco, Milan, Italy. Tiziana Varisco and Olivia Casati: Department of Paediatrics, Desio Hospital, ASST Brianza, Monza, Italy. Alberto Fabio Podestà and Maddalena Gibelli: Department of Paediatrics and Neonatology, San Carlo Borromeo Hospital, ASST Santi Paolo e Carlo, Milan, Italy. Stefano Martinelli and Roberta Restelli: Neonatology and Neonatal Intensive Care Unit, Niguarda Hospital, Milan, Italy. Laura Maria Pogliani and Roberta Agistri: Department of Paediatrics, Neonatology and Neonatal Pathology, Legnano Hospital, ASST Ovest MI, Milan, Italy. Marco Giuseppe Nedbal and Paolo Vaglia: Department of Paediatrics and Neonatology, Gallarate Hospital, ASST Valleolona, Milan, Italy. Chryssoula Tzialla and Luisa Magnani: Department of Paediatrics and Neonatology, Oltrepò Pavese Hospital, ASST Pavia, Pavia, Italy. Elena Sala and Laura Lorioli: Department of Neonatal Pathology, ASST Papa Giovanni XXIII, Bergamo, Italy. Giuseppe Banderali and Diana Ghisleni, Clinical Department of Neonatology, San Paolo Hospital, ASST Santi Paolo e Carlo, Milan, Italy. Bruno Drera and Marta Frittoli: Department of Neonatal Pathology, ASST Cremona, Cremona, Italy. Francesca Lizzoli and Marta Bellini: Department of Paediatrics, Neonatology and Neonatal Pathology, Magenta Hospital, ASST Ovest Milanese, Milan, Italy. Paola Bruni and Ilaria Giulini, Department of Paediatrics, ASST Melegnano-Martesana, Milan, Italy. Valentina Benedetti and Valentina Polimeni: Department Neonatology and Neonatal Intensive Care Unit, IRCCS Ca' Granda Ospedale Maggiore Policlinico, Milan, Italy. Nadia Salvoni and Masotina Raffaele: Department Neonatology and Neonatal Pathology, Sacco Hospital, ASST Fatebenefratelli-Sacco, Milan, Italy. Cristina Bellan and Roberto Bottino: Department Neonatology and Neonatal Intensive Care Unit, Bolognini Hospital, ASST Bergamo Est, Bergamo, Italy. Graziano Barera and Antonella Poloniato: Neonatal Unit, San Raffaele Scientific Institute, Milan, Italy. Marta Odoni and Ilaria Dalla Verde: Department of Paediatrics and Neonatology, Policlinico San Pietro, Gruppo Ospedaliero San Donato, Bergamo, Italy. Massimo Agosti and Angela Bossi: Department of Paediatrics, Neonatology and Neonatal Intensive Care Unit, ASST Settelaghi, Varese, Italy. Anna Tosi and Anna Elisabetta Bussolini: Department of Paediatrics, Tradate Hospital, ASST Settelaghi, Varese, Italy. Francesco Maria Risso and Vania Spinoni: Department of Neonatology and Neonatal Intensive Care Unit, Children Hospital, ASST Spedali Civili, Brescia, Italy. Nicola Altamura and Patrizia Ballista: Department of Paediatrics and Neonatology, Sesto San Giovanni Hospital, ASST Nord Milano, Milan, Italy. Silvia Di Chio and Luciana Pagani: Department of Neonatal Pathology and Neonatal Intensive Care Unit, Macedonio Melloni Hospital, ASST Fatebenefratelli-Sacco, Milan, Milano. Lidia Decembrino and Michela Grignani: Department of Paediatrics and Neonatology, Vigevano Hospital, ASST Pavia, Pavia, Italy. Grazia Morandi and Valeria Angela Fasolato: Department of Neonatology and Neonatal Intensive Care Unit, Carlo Poma Hospital, ASST Mantova, Mantova, Italy. Lorella Rossi and Emilio Palumbo: Department of Paediatrics and Neonatology, Eugenio Morelli Hospital, ATS Montagna, Sondrio, Italy. Alessandro Lepore and Maria Forestieri: Department of Paediatrics and Neonatology, Busto Arsizio Hospital, ASST Valleolona, Varese, Italy. Stefano Ghirardello and Elisa Civardi: Department of Neonatal Pathology and Neonatal Intensive Care Unit, San Matteo Research Hospital, Pavia, Italy. Paolo Adamoli: Department of Paediatrics, Moriggia Pelascini Hospital, Gravedona, Italy. Roberta Giacchero: Department of Paediatrics and Neonatology, ASST Lodi, Lodi, Italy. Giovanni Traina: Department of Paediatrics, ASST-Melegnano Martesana, Melzo, Italy. Salvatore Barberi: Department of Paediatrics, Rho Hospital, ASST-Rhodense, RHO, Milan, Italy. Patrizia Calzi and Fenesia Pedace: Department of Neonatology, Carate Brianza Hospital, ASST Brianza, Carate, Italy. Marco Sala: Department of Paediatrics, Vimercate Hospital, Vimercate, Italy.

The authors apologize for this error and state that this does not change the scientific conclusions of the article in any way. The original article has been updated.

